# Supplementary figures and images for: Genome-wide identification of the bHLH transcription factor family in Rosa persica and response to low-temperature stress
Source: PeerJ. 2024 Jan 3;12:e16568. doi: 10.7717/peerj.16568 (PMC10771085; doi:10.7717/peerj.16568)

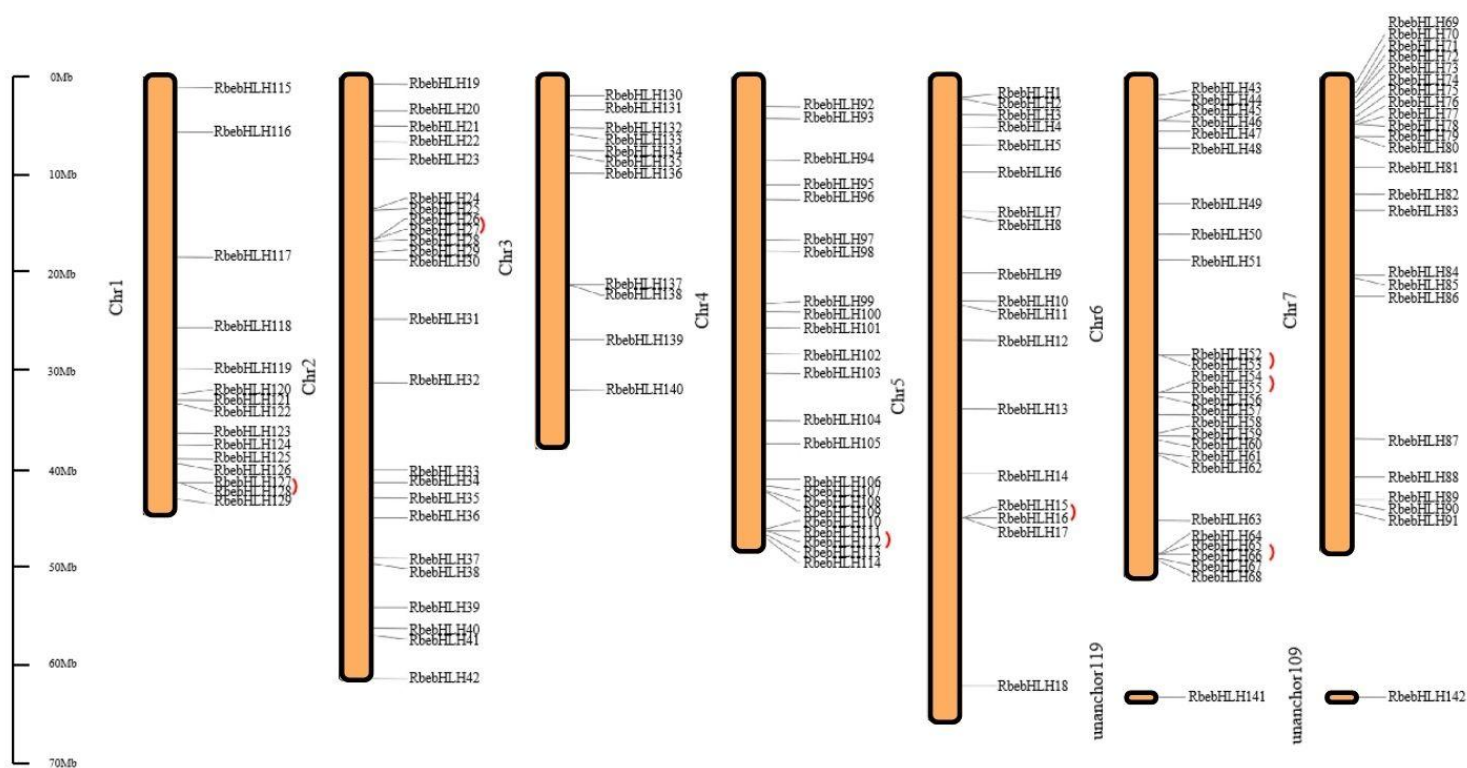

Supplement: Supplemental Information 1 [file peerj-12-16568-s001.pdf]

Motif1

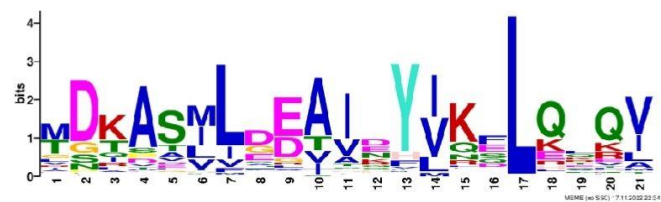

Motif6

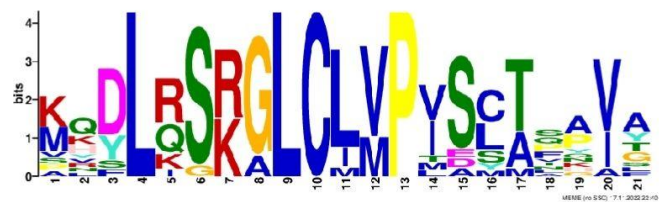

Motif2

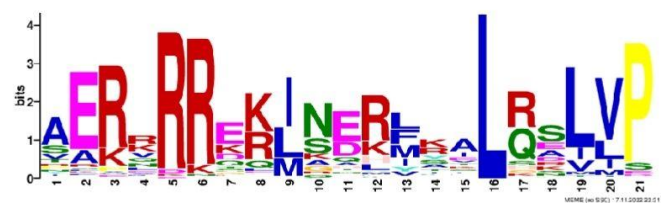

Motif7

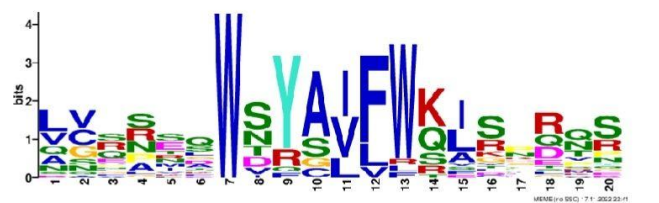

Motif3

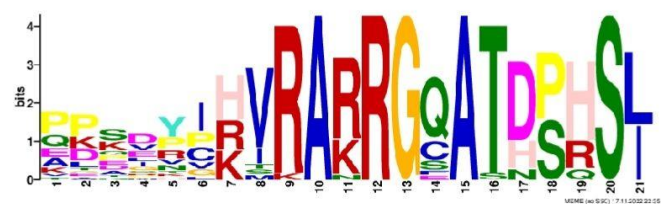

Motif8

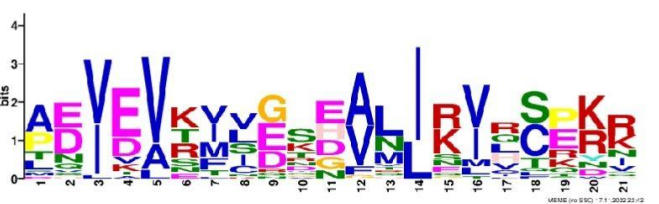

Motif4

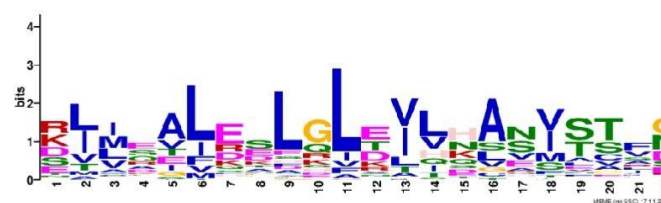

Motif9

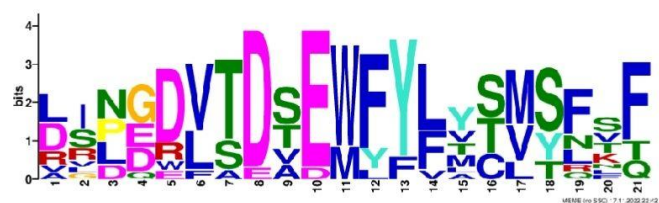

Motif5

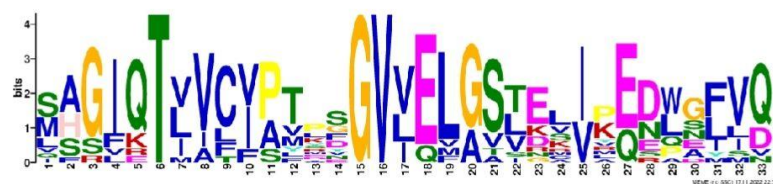

Motif10

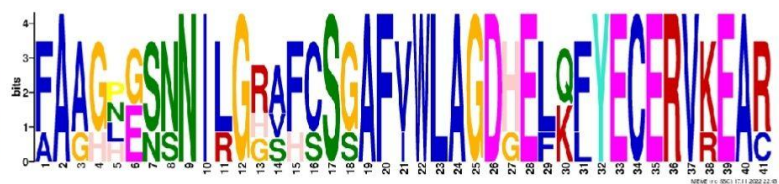

Supplement: Supplemental Information 2 [file peerj-12-16568-s002.pdf]

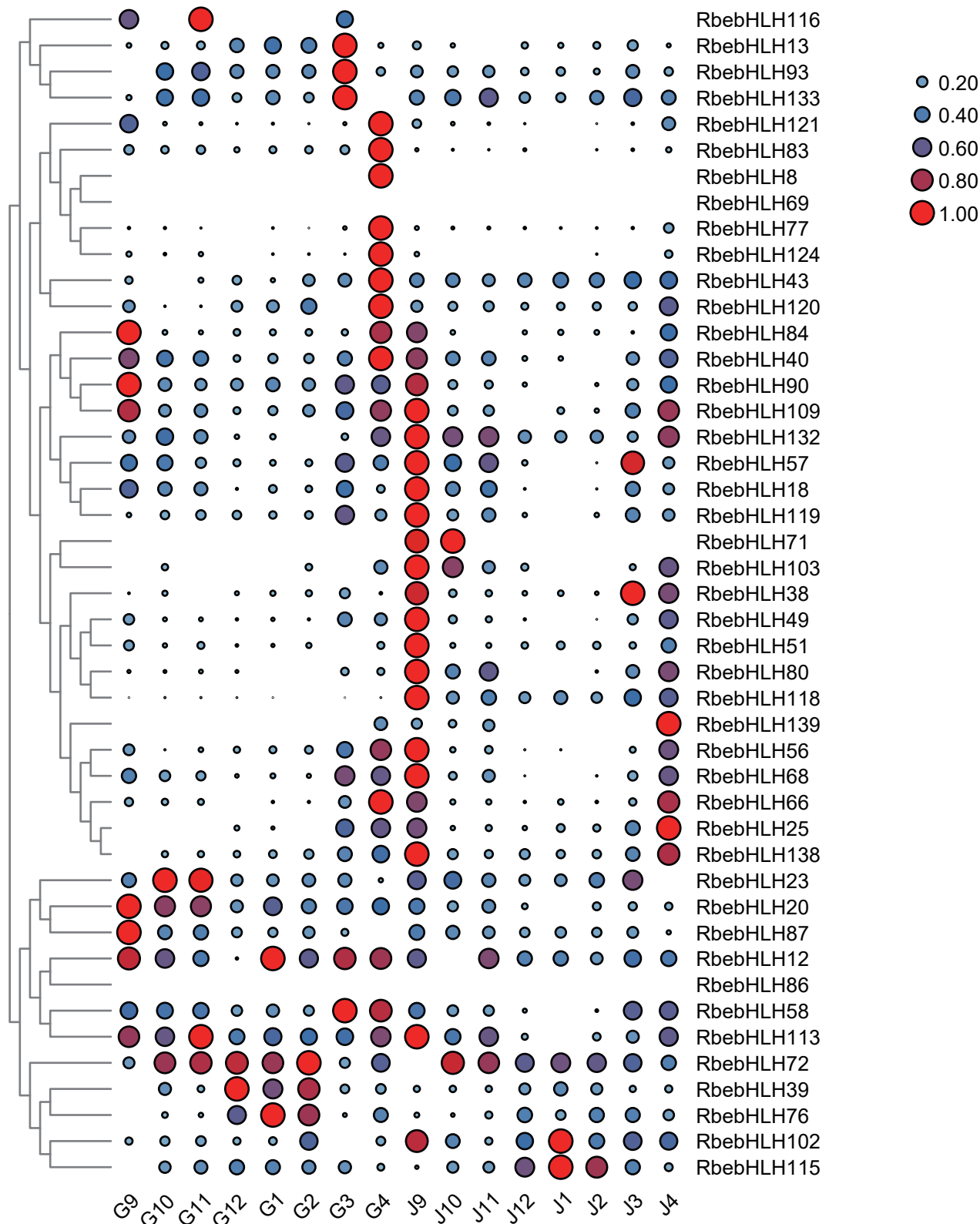

Supplement: Supplemental Information 3 — G: root; J: stem. Numbers indicate months and colors represent expression levels, with red representing high expression and blue representing low expression. The size of the circles is proportional to the expression of the gene. [file peerj-12-16568-s003.pdf]
